# Biofilms as agents of Ediacara-style fossilization

**DOI:** 10.1038/s41598-022-12473-1

**Published:** 2022-05-23

**Authors:** Silvina Slagter, Weiduo Hao, Noah J. Planavsky, Kurt O. Konhauser, Lidya G. Tarhan

**Affiliations:** 1 Department of Earth and Planetary Sciences, Yale University, New Haven, CT 06511 USA; 2 Department of Earth and Atmospheric Sciences, University of Alberta, Edmonton, AB T6G 2E3 Canada

**Keywords:** Palaeontology, Precambrian geology, Geochemistry

## Abstract

Earth’s earliest fossils of complex macroscopic life are recorded in Ediacaran-aged siliciclastic deposits as exceptionally well-preserved three-dimensional casts and molds, known as “Ediacara-style” preservation. Ediacara-style fossil assemblages commonly include both macrofossils of the enigmatic Ediacara Biota and associated textural impressions attributed to microbial matgrounds that were integral to the ecology of Ediacara communities. Here, we use an experimental approach to interrogate to what extent the presence of mat-forming microorganisms was likewise critical to the Ediacara-style fossilization of these soft-bodied organisms. We find evidence that biofilms can play an instrumental role in fostering fossilization. Rapid silica precipitation associated with macroorganism tissues is enhanced in the presence of mat- and biofilm-forming microorganisms. These results indicate that the occurrence of microbial mats and biofilms may have strongly shaped the preservational window for Ediacara-style fossils associated with early diagenetic silica cements, and therefore influenced the distribution and palaeoecological interpretation of the Ediacara Biota fossil record.

## Introduction

Fossils of the Ediacara Biota record the earliest ecosystems of macroscopic, architecturally complex multicellular organisms, including clear evidence for animals^1^. The majority of Ediacara fossil assemblages are preserved in the “Ediacara style,” in which these soft-bodied organisms are replicated as casts and molds within relatively coarse siliciclastic rocks such as sandstones and siltstones. These fossiliferous impressions encompass a variety of modes, including bed-top (epirelief), bed-sole (hyporelief) and bed-interior (endorelief) preservation of features interpreted to variably reflect these organisms’ external or internal morphology, including both their upper and lower surfaces. Diverse examples of Ediacara-style preservation have historically been described using a variety of names, such as “Flinders-style”, “Conception-style” and “Nama-style” preservation^1^, that commonly reference particular localities or units in which they were first described. However, recent work has highlighted that individual units—as well as individual facies, bedding planes and taxa—may be characterized by multiple modes of Ediacara-style preservation (e.g., Ref.^2^). Recent studies have also revealed a wider range of variants of Ediacara-style preservation not included under older nomenclatural schemes, such as fossils interpreted to record hyporelief rather than endorelief preservation of internal molds^2^, part-counterpart moldic preservation^3^, hyporelief external molds of organisms’ lower surfaces^4^, negative-relief casts of internal molds^5^ and composites of both internal and external molds^2^. Fossil assemblages characterized by both individual and multiples of these modes occur in various geologic units of Proterozoic through lower Palaeozoic age (ca. 2500 to 360 million years ago)^6^. We hereafter refer to these modes collectively as Ediacara-style preservation. The phylogenetic affinities and functional ecologies of Ediacara organisms have been extensively debated, due largely to the enigmatic morphology of most Ediacara taxa and limited understanding of what mechanisms may have facilitated this unusual style of fossilization. Resolving the processes responsible for Ediacara-style fossilization is essential to accurately reconstruct Ediacara community structure, the distribution of Ediacara taxa in time and space and, more broadly, the significance of the Ediacara Biota in the emergence and subsequent evolutionary trajectory of complex life on Earth.

**Figure 1:**
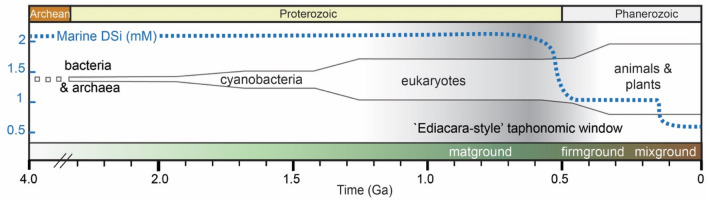
Temporal variation in marine dissolved silica (DSi) levels, in the context of other major environmental and biotic changes over this interval. Representation of inferred temporal changes in DSi follows Ref.^25^. Changes in the relative abundance of seafloor substrate type (matgrounds, firmgrounds, and mixgrounds)^27^ are indicated in the lower bar. The spindle plot represents the evolutionary diversification of major clades (modified after Ref.^73^). The shaded grey vertical line shows the estimated duration of the Ediacara-style taphonomic window^6^.

A wide range of competing hypotheses have been proposed regarding what taphonomic (i.e., preservational) pathways may have been integral to Ediacara-style fossilization, including resistant external tissues^7,8^, rheological differences within the sediments that host fossils^9^ or authigenic mineralization^10–17^. Other factors, such as rapid burial and limited bioturbation or macrofaunal scavenging, may have contributed to the formation of Ediacara-style fossil deposits, as they did to Proterozoic and Phanerozoic deposits hosting other modes of exceptional soft-tissue fossilization^18,19^. However, neither rapid burial nor limited bioturbation or scavenging are unique to the Ediacaran Period or to Ediacara-style fossil deposits, and it is therefore unlikely that these factors were principal drivers of Ediacara-style fossilization. Similarly, preservation of Ediacara Biota taxa in other Ediacaran deposits characterized by other taphonomic modes^20^ (including modes extensively documented in Phanerozoic deposits), as well as the occurrence of biostratinomically deformed Ediacara soft tissues^21^, suggest that Ediacara Biota organisms did not possess unusually strong integuments nor were these essential for their fossilization. Ediacara fossilization by means of authigenic mineralization—specifically, the potential importance of rapid precipitation of iron minerals^10–12^, clays^13–15^, or silica^6,16^ in facilitating capture of soft-tissue features prior to carcass collapse and degradation—has of recent years been a topic of particular interest and contention. Although debate has continued regarding the syngeneity and relative importance of specific minerals^6,22–24^, both geological^6^ and experimental^16^ recent work has indicated that high dissolved silica (DSi) concentrations in the Ediacaran oceans^25,26^—potentially an order of magnitude higher than present-day marine dissolved silica levels—may have created a taphonomic window that favoured the moldic preservation of soft tissues via early diagenetic precipitation of silica cements, even from undersaturated solutions (Fig. 1). This early cementation model suggests that silica precipitated from DSi-enriched seawater onto the organic substrates provided by shallowly buried Ediacara ecosystems, as well as in the surrounding pore spaces^6^. Although Ediacara Biota taxa are represented by a range of preservational modes^20,27^, the majority are preserved moldically in the Ediacara style, and silica cementation has emerged as a likely fossilization pathway for the Ediacara Member, the most diverse of these Lagerstätten and one which includes multiple variants of moldic, Ediacara-style preservation^2,6^.

Ediacara-style fossilization is not only distinctive of Ediacara Biota macroorganisms but also of microbial mats and biofilms or “matgrounds” that carpeted the Ediacaran seafloor and are similarly recorded as a variety of moldic textures, known as textured organic surfaces (TOS), in Ediacara fossil assemblages^28^. The characteristic spatial and ecological association of Ediacara macrofossils and microbial mat and biofilm textures across a wide range of Ediacara-style fossil deposits^29–31^ has led to the suggestion that matgrounds, in addition to fostering the habitability of the Ediacara seafloor^32–39^ and shaping the ecology of Ediacara communities^28,40–42^, may have also played a critical role in their fossilization^5,43–49^. Previous work has highlighted that bacteria may readily undergo silicification via Bitter Springs-style fossilization (i.e., permineralization of filamentous and coccoid microorganisms in cherts^50–53^). However, direct constraints on the contributions of microbial mats and biofilms to Ediacara-style fossilization—and thus the fossil record of the earliest animal communities—are lacking. Accordingly, we describe here the first experimental replication of moldic preservation of soft-bodied animals in quartzose sand, i.e., Ediacara-style preservation, under high-DSi conditions and in the presence of microbial mat-forming organisms.

## Results

**Figure 2:**
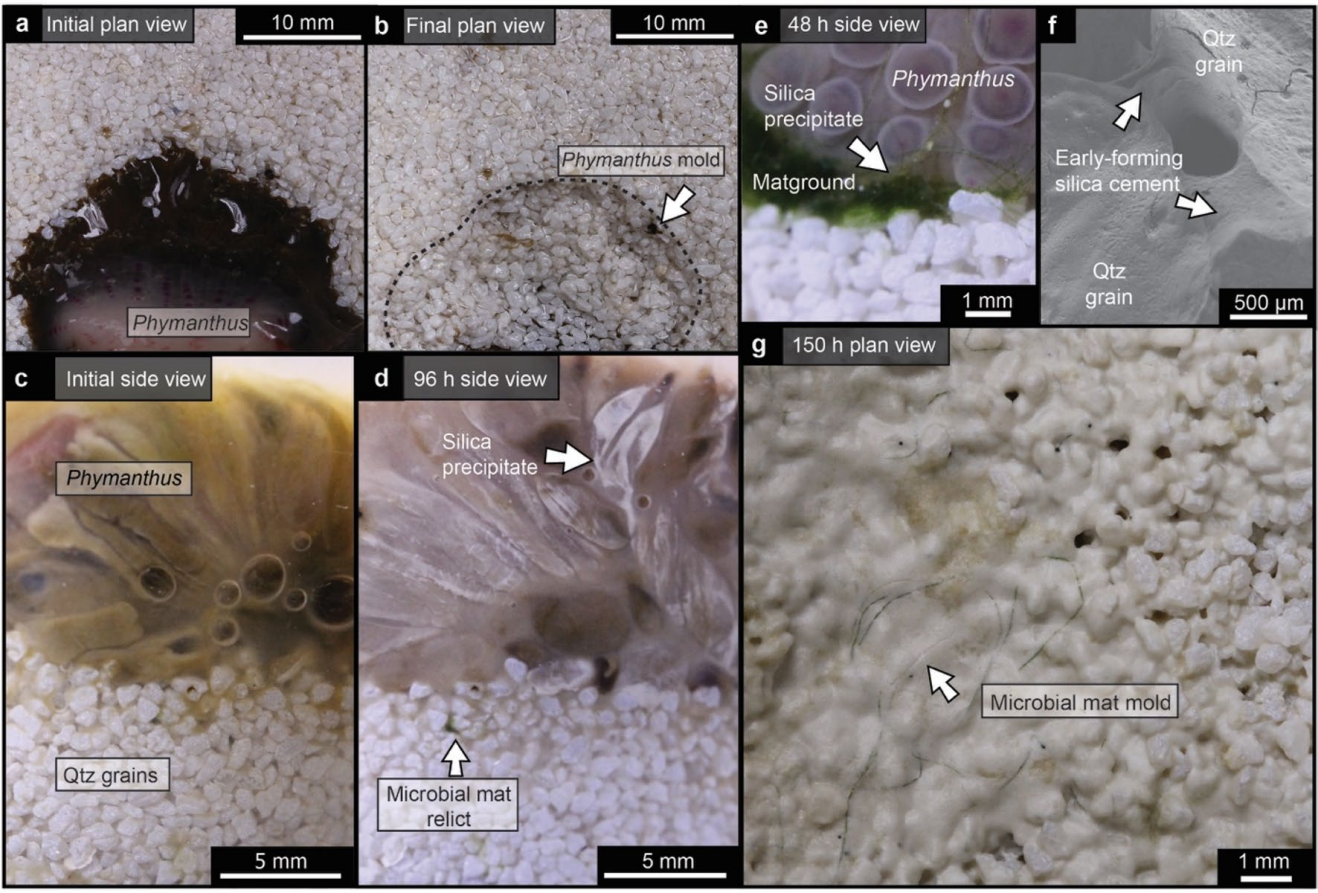
Ediacara-style experiments through time. (**a**,**b**) Plan-view photographs of a representative fossilization experiment conducted with *Phymanthus*, *Anabaena, Fischerella, Spirogyra*, and *Vaucheria*, taken at the start (0 h; **a**) and end (150 h; **b**) of the experiment (experiment 11, Table 1). (**c**–**e**) Cross-section photographs taken at distinct intervals throughout experiment 11, showing *Phymanthus* shortly following the start of the experiment (0 h; **c**); silica precipitates on the exterior of *Phymanthus* tentacles after 96 h (**d**); and silica precipitates associated with both *Phymanthus* and biofilms after 48 h (**e**). (**f**) SEM secondary electron image showing the presence of early-forming silica cements between sand grains after 72 h in experiment 11. (**g**) Plan view of an experiment with biofilms (experiment 10, Table 1) that, following degradation of organic matter, was characterized by molds of microbial filaments, associated with extensive (including inter-granular) silica precipitates.

To test to what extent the presence of microbial mats might influence Ediacara-style preservation by means of silica cementation, we performed taphonomic (i.e., controlled decay) experiments with initial DSi concentrations of 2 mM, as an approximation of Precambrian seawater^25,26^. These taphonomic experiments included the anemone *Phymanthus*, both with and without accompanying cyanobacterial and algal aggregates (cyanobacteria *Anabaena* and *Fischerella*; green algae *Vaucheria* and *Spirogyra*), in association with a quartzose sand substrate (Table 1). Control experiments without high DSi concentrations experienced neither silica precipitation nor moldic preservation. Experiments performed under initially high-DSi conditions, but without biofilms, produced molds that were comparatively poorer in fidelity (i.e., fewer morphological details captured; see Supplementary Fig. S1). By contrast, experiments involving *Phymanthus* and mat-forming organisms under high initial DSi concentrations experienced a significant drawdown of DSi levels, yielding concentrations as low as 0.2 mM after 150 h. Visible silica precipitates along *Phymanthus* were observed after 110 h, whereas silica precipitates were observed along microbial aggregate interfaces, as well as in surrounding inter-granular pore spaces, after only 72 h (Fig. 2). Scanning electron microscopy–energy dispersive X-ray spectroscopy (SEM–EDS) analyses indicate that these precipitates form as silica nanospheres (Fig. S2). DSi measurements taken at the termination of each experiment indicate that experiments performed with biofilms were characterized, on average, by greater DSi drawdown than experiments performed with *Phymanthus* alone (Table S1). Final silica concentrations between experiments with and without biofilms are statistically significantly different (Mann Whitney U test *p* value < 0.05; Table S2). Further, the presence of biofilms appeared to directly influence the locus of silica precipitation, which was enhanced in the vicinity of the biofilms, resulting in negative-relief impressions in the quartzose sandy substrate underlying both macro- and microorganisms (Fig. 2b,g). Experimental samples associated with moldic impressions were, by the termination of each experiment, characterized by low organic carbon concentrations as a result of substantial degradation of macroorganism and microorganism organic matter (Table S3).

**Table 1:** Experimental setup.

Experiment		Initial DSi (mM)	Sand (g)	*Phymanthus* (g)	Algae, cyanobacteria (g)	MOPS** (ml)
1	no DSi + *Phymanthus*	0	–	16.29	–	2
2	no DSi + *Phymanthus* + sand	0	50	28.08	–	2
3	DSi + sand	2	50	–	–	2
4	DSi + *Fischerella* + sand	2	50	–	10	2
5	DSi + *Anabaena* + sand	2	50	–	10	2
6	DSi + *Vaucheria* + sand	2	50	–	10	2
7	DSi + *Spirogyra* + sand	2	50	–	10	2
8	DSi + *Phymanthus*	2	–	15.23	–	2
9	DSi + *Phymanthus* + sand	2	50	12.33	–	2
10	DSi + sand + biofilm*	2	50	–	10	2
11	DSi + *Phymanthus* + sand + biofilm*	2	50	4.22	10	2

*DSi* dissolved silica. Noted masses of Phymanthus are for the first run of each experiment.

Biofilm refers to equal proportions of *Fischerella, Anabaena, Vaucheria,* and *Spirogyra.*

**3-(N-morpholino)propanesulfonic acid.

**Figure 3:**
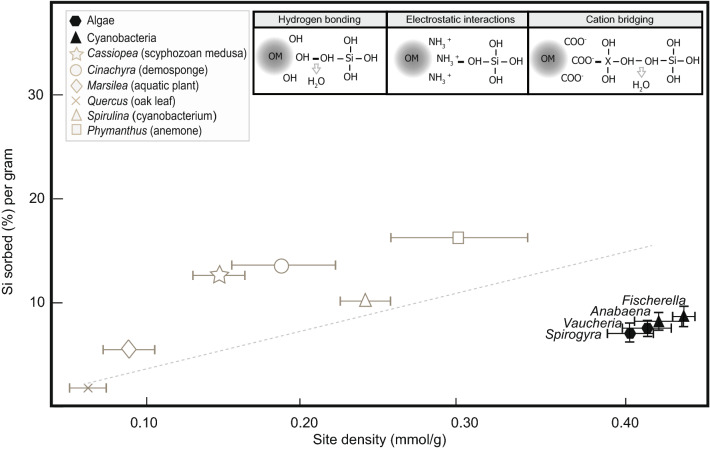
Potentiometric titration results. Mean reactive site density (mmol/g) for experimental organisms, plotted against Si absorbed, measured at the end of each experiment (150 h), and normalized to the average initial weight of each specimen. Error bars represent the standard deviation. Illustrations along the top of the panel represent the molecular bonding of silica to (from left to right) hydroxyl, amino, and carboxyl groups of organic matter (OM) (modified from Ref.^54^). “X” represents a metal cation. Gray symbols denote data from Ref.^16^. Black symbols represent new data produced for this study. Line denotes best-fit approximation of all these data (y = 83.694x−10.604; R^2^ = 0.7795).

**Figure 4:**
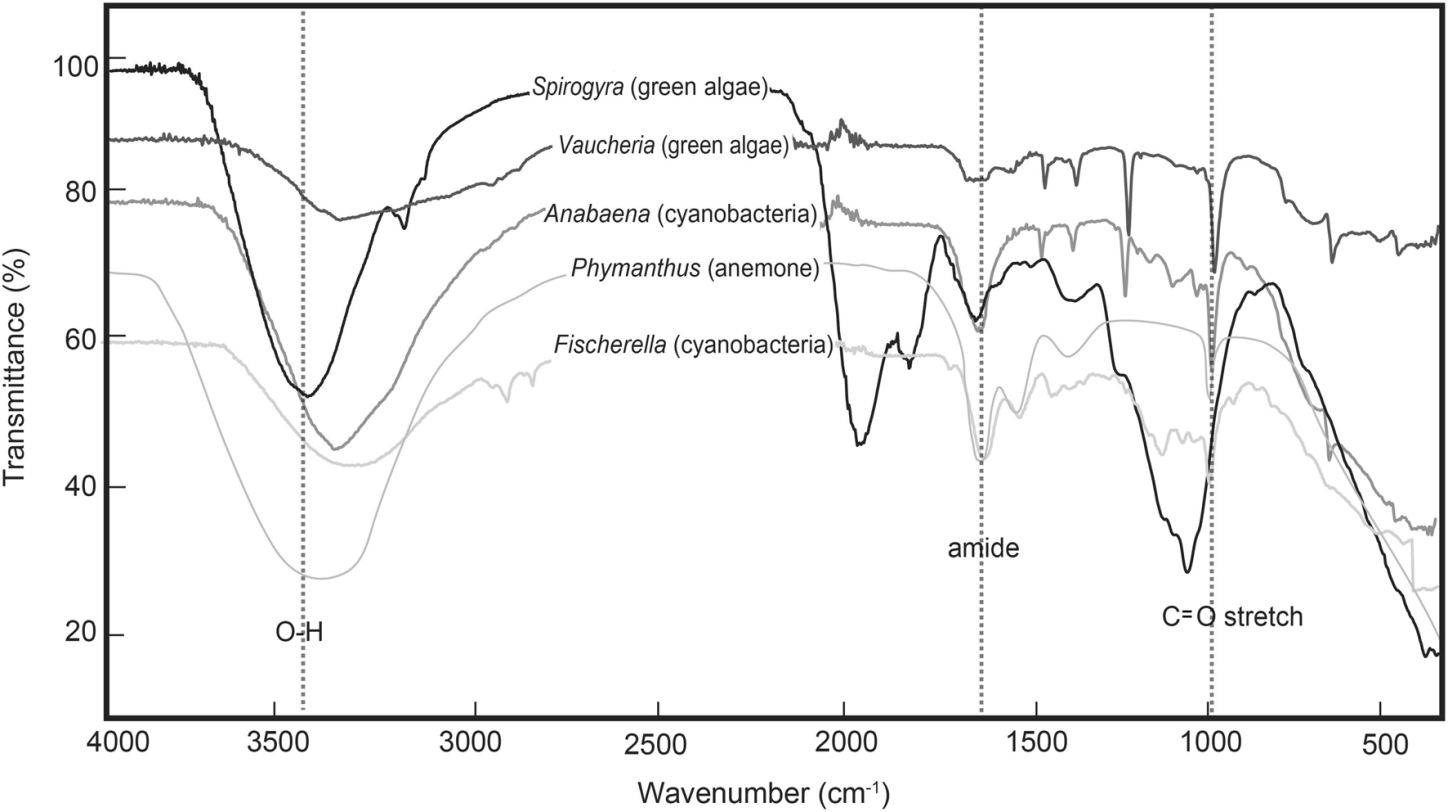
Fourier-transform infrared (FTIR) results. Spectra of specimens at the start of experiments, indicating the presence of O–H bonds, amide bonds, and C=O stretch in the organic tissues of these taxa, and which provide highly reactive sites for silicification.

In order to investigate what factors, on a molecular level, were responsible for the greater silicification and higher-fidelity (more anatomically detailed) moldic preservation we observed under silica-enriched solutions and in the presence of biofilms, we also analysed experimental samples using potentiometric titrations and Fourier transform infrared spectroscopy (FTIR). Potentiometric titration data indicate that the algal and cyanobacterial taxa populating these mats are characterized by higher surface reactivities than the marine invertebrate animals investigated in this and previous studies^16^, including scyphozoan medusae, anemones, and sponges (Fig. 3). Furthermore, experiments including biofilms resulted in higher silica sorbed per gram of biomass than experiments without mat-forming microorganisms (Table S4). Acid–base titration results were best fit by a three-site protonation model corresponding to three main functional groups (denoted as ≡LH, ≡XH, and ≡MH), interpreted as carboxyl, phosphoryl, and amino or hydroxyl groups, respectively (Figs. S3,S4; Table S5). The presence of carboxyl and amino groups in the biofilms (in addition to amino and hydroxyl groups in the macroorganisms) was also verified via FTIR (Fig. 4). From these data, we infer that carboxyl, hydroxyl, and amino functional groups within these organisms’ tissues and cell walls provided adsorption sites that were highly reactive to DSi and directly mediated silica precipitation. Previous studies have indicated that hydrogen bonding between silicic acid and hydroxyl groups associated with organic surfaces^54,55^ favours the formation of silanol (Si–OH)^56^. Other mechanisms, such as electrostatic attraction of Si by amino groups on organic substrates^57,58^ and cation bridging between carboxyl and negatively charged silica species, may also result in the bonding of silica to organic surfaces^59^ (Fig. 4). These observations provide evidence that the presence of microbial (cyanobacterial and algal) biofilms could have fostered Ediacara-style fossilization as well as yielding critical insights regarding the molecular pathways responsible for this distinctive mode of preservation.

## Discussion

Our observations are consistent with reports of exceptionally preserved biofilm textures—and, in some instances, potential cyanobacterial sheaths^60–64^—in quartzose sandstones, including Ediacara-style fossil assemblages^32,65–69^, and which have long formed the basis for the inferred ecological association between Ediacara macroorganisms and matgrounds. We interpret that the high reactivity of the biofilms on and in which Ediacara macroorganisms lived—and with which they were rapidly buried—played a critical role in enhancing silica precipitation in the surrounding sandy sediments. Initial cement precipitation would, in this framework, have fostered additional cement growth, on both organic substrates and in intergranular spaces. This, in turn, could have driven rapid and extensive cementation of intergranular pore spaces with amorphous silica. Importantly, the highly silica-reactive nature of matground communities and the proximity of buried macroorganisms to overlying bottom waters suggest that silica-enriched pore waters (supplied by diffusion from overlying silica-enriched bottom waters) could have facilitated cementation of both the lower (matground-proximal and thus more highly reactive) and upper (bottom water-proximal and thus requiring lower diffusional transport distances) surfaces of benthic Ediacara organisms. High dissolved silica levels could therefore have mediated continuous precipitation of silica cements at a variety of loci within seafloor sediments, providing an explanation for the variety of moldic modes recorded by Ediacara-style fossil assemblages. Rapid silica cementation assisted by proximity to highly reactive matgrounds would, moreover, have facilitated the preservation of both macrofossils and associated mat-forming communities as sandstone molds (e.g., preservation of matgrounds as TOS). Given the variety of mat-related ecologies characteristic of Ediacara macroorganisms and regions of seafloor sediments that biofilms and matgrounds inhabited, we interpret this to reflect enhanced reactivity of the entire substrate where biofilms (and macroorganism carcasses) were present. Our experiments also demonstrate that silica precipitates persist even after the organisms have entirely decayed, as is characteristic of Ediacara-style moldic fossil assemblages, which typically lack organic remnants of organism tissues. The greater propensity of macroorganism soft tissues to be moldically preserved via silica cementation in the presence of microbial populations further suggests that environmental parameters—rather than anomalously robust macroorganism tissues^7,8^—were responsible for instances of Ediacara-style fossilization associated with early diagenetic silica cements. This provides critical support for the idea that Ediacara-style preservation can offer accurate snapshots of early macrofaunal ecosystems, but also highlights that matground substrates may have shaped the fossilization process.

An important consideration stemming from this experimental work is that the spatial extent and ecological maturity of matground- and biofilm-associated communities likely influenced the fossilization potential of Ediacara macroorganisms. Under higher-DSi conditions, but in the absence or relative paucity of microbial mats (e.g., as in certain lower Palaeozoic instances of Ediacara-style fossilization)^6^, moldic preservation of macroorganisms via silicification may still have occurred^16^, but potentially with lower fidelity than seafloor ecosystems associated with well-developed matgrounds. Consequently, although high-DSi ocean waters would have provided a global-scale potential for Ediacara-style fossilization linked to early silica cementation, local taphonomic factors—such as the presence or absence of well-developed microbial matgrounds—likely played a secondary but critical role in shaping fossilization potential and fidelity. Extensive microbial matgrounds and biofilms in regions of the Ediacaran seafloor may therefore have contributed to instances of exceptional preservation of populous Ediacara communities with anomalously high (relative to Phanerozoic analogues) alpha and beta diversity (cf.^32,70^). But, in light of the heterogeneous distribution of Ediacara matgrounds (e.g.^11,32^) and the likely variability in matground-mediated fossilization, caution should be exercised when making broad ecological and evolutionary inferences from individual Ediacara-style fossil deposits.

Our experimental investigation of animal soft-tissue moldic preservation in the presence of silica-enriched seawater, a sandy substrate, and biofilms has yielded new insights into the pathways and mechanisms responsible for Ediacara-style fossilization of Earth’s earliest animal communities, particularly Ediacara-style assemblages associated with early diagenetic silica cements. These findings highlight that Ediacaran microbial mats not only played an integral ecological role in the development of complex Ediacara macrofaunal communities^32,70^ but may have also contributed directly to Ediacara-style fossilization by serving as highly reactive loci for silica precipitation. We demonstrate that silica precipitation onto both biofilms and macroorganism tissues can easily occur under dissolved Si concentrations and pH values reconstructed for Ediacaran seawater^71^, and can even proceed after dissolved silica levels drop below the thermodynamic thresholds traditionally considered necessary to facilitate precipitation of amorphous silica^16,72^. The association of numerous Ediacara Biota fossil assemblages with textural and sedimentary evidence for widespread microbial mats indicates that this association may have been taphonomically, as well as ecologically, critical. In sum, the Ediacaran—with its combination of complex macrofaunal seafloor communities and well-developed matgrounds—may have been uniquely well-suited to fostering a taphonomic window for exceptional, Ediacara-style fossilization.

## Materials and methods

### Experimental setup

Specimens of the anemone *Phymanthus crucifer,* cyanobacteria *Anabaena* and *Fischerella,* and green algae *Spirogyra* and *Vaucheria* were purchased from the Carolina Biological Supply Company (Burlington, NC, USA). Experimental substrates of pure quartz sand of 0–2 φ (0.2–0.8 mm diameter) were also purchased from Carolina Biological Supply Company (Burlington, NC, USA), and sterilized prior to use by autoclaving at 121 °C for 15 min. All experiments were performed in triplicate and conducted in tightly capped sterile plastic culture jars at a constant temperature of 25 °C. The experiments were initiated by the inoculation of euthanized animals and/or cyanobacterial and algal cultures onto the surface of the sand, followed by the addition of Si-rich artificial seawater with Na_2_SiO_3_.9H_2_0 (Table S6), with a concentration of 2 mM chosen to represent Ediacaran seawater^25,26^. An additional burial layer of sand was not included in these experiments, due to the challenges associated with sampling, photographing and observing morphological changes without disturbing experimental conditions and due to the potential impact on diffusion, chemical gradients and reactivity that would have been imposed by mechanisms commonly used for removal of experimental burial sands (e.g., plastic film; Ref.^44,45^). The animals' care was in accordance with institutional guidelines. The solution pH was immediately adjusted by the addition of 1 N HCl. A pH value of 7.8 was maintained for the duration of the experiments by the addition of 1 M 3-(N-morpholino) propanesulfonic acid (MOPS) buffer (Table 1). After 150 h, samples were UV-irradiated, and hydrogen peroxide was then added to degrade any remaining organic matter. To track changes in solution chemistry associated with organic decay, small aliquots of solution (2 mL) were sampled by syringe at sequential timed intervals throughout the experiment and stored in tightly capped polyethylene containers to analyze dissolved silica concentrations. Solution pH was measured using a Thermo Scientific™ Orion™ PerpHecT™ ROSS™ Combination pH Micro Electrode, calibrated with pH buffers of 4.0, 7.0, 10.0 before each analysis. At each time point, substrate samples were transferred to mounts for observations under a scanning electron microscope, and additional samples were taken for carbon content both before and after UV-irradiation and addition of peroxide. From these, splits were freeze-dried for spectroscopic analyses.

### Dissolved silica concentrations

Silica concentrations were measured by the molybdate blue method with metol as the reducing agent at 812 nm using a UV/VIS spectrophotometer^74^. All samples were measured with three replicates with less than 5% (1σ) error in absorption values.

### Carbon content

Bulk weight percent C measurements were performed at the Yale Analytical Stable Isotope Center. Samples were analyzed with a Costech ECS 4010 Elemental Analyzer coupled to a Thermo Delta Plus Advantage isotope ratio mass spectrometer. Data were corrected using internal glutamic acid reference materials YGA and CN2, which were calibrated to V-PDB using United States Geological Survey (USGS) glutamic acid. In addition, an internal reference (trout) served as a quality control check. The precision for each run was within ± 0.2 ‰ based on the performance of the internal reference materials.

### Fourier transform infrared (FTIR) spectroscopy

FTIR was used to determine the dominant types of organic ligands associated with experimental organic tissues and precipitates, and to assist in assigning functional group identities to the sites modelled from potentiometric titration data. Samples for FTIR were freeze-dried for 24 h prior to analysis. The freeze-dried samples were then analysed using a Thermo Nicolet FTIR Spectrometer 6700 and a Shimadzu IR Tracer-100 at the Yale Chemical and Biophysical Instrumentation Center. Three scans were collected for each spectrum over the range of 40.00–500 cm^−1^ in transmittance mode with a resolution of 4.0 cm^−1^ to 0.25 cm^-1^. Background spectra were also collected and subtracted from the sample’s spectra with the Fityk software.

### Scanning electron microscopy (SEM)

SEM and energy-dispersive x-ray spectroscopy (EDS) analyses were performed at 3–10 kV with a Hitachi SU7000 scanning electron microscope in the Department of Earth and Planetary Sciences at Yale University. Organic tissue samples were previously fixed in 4% paraformaldehyde, dehydrated through a graded ethanol series, air dried, and then coated with 6 nm platinum for SEM–EDS analyses.

### Statistical analyses

Shapiro-Wilks tests of silica concentration values from both experiments with *Phymanthus* and those with *Phymanthus* and biofilms (n = 42) indicate that these data are not normally distributed (p < 0.05). The Mann–Whitney U test was therefore performed to verify whether these two groups are significantly different. All tests indicate statistically robust (p < 0.05) significant differences between median Si concentrations in experiments with and without biofilms (Table S2).

### Surface reactivity measurements

Potentiometric acid–base titrations were performed at the Department of Earth and Atmospheric Sciences at the University of Alberta. Analyses were performed for the organic tissues of each experimental taxon (5 samples of each) to determine their reactivities (i.e., potential for protonation). Before each titration, the pH electrode was calibrated using commercial pH buffers (Thermo Fisher Scientific; pH 4.0, 7.0, 10.0). For each titration, organic tissues were suspended in a 0.56 M NaCl solution. The suspension was then bubbled for 30 min with N_2_(g) to ensure the solution was devoid of CO_2_; during titrations, the experimental apparatus remained sealed and was continuously bubbled with N_2_ to prevent CO_2_ from entering the system. Each organic tissue sample was titrated over a pH range of 3.0 to 10.5. Initially, a small volume of 0.1 M nitric acid (HNO_3_, ACS-certified, Fisher Scientific) was added to bring the solution pH down to 3.0, and then 0.1 M sodium hydroxide (NaOH, ACS-certified, Fisher Scientific) solution was incrementally added to bring the pH up to 10.5 (forward titration). To test the hysteresis of the samples, a backward titration was performed after each forward titration by adding acid to decrease the solution pH from 10.5 to 3.0. After each aliquot of acid or base was added, the corresponding pH changes were recorded. The pH was considered stable only after the electrode achieved a reading of 12 mV/min. A blank titration, without the addition of biomass, was performed for electrolyte solutions for each of the titrations performed.

### Surface complexation model

Surface complexation modelling was performed on the basis of potentiometric acid–base titration results of organic tissue samples of the studied organisms. Three types of surface functional groups (≡LH, ≡XH, ≡MH) were identified as the model to represent the most proton-reactive groups. The protonation behaviours of these three surface sites are modelled as follows:

R1 $$\equiv {\text{L}}^{ - } + {\text{ H}}^{ + } \leftrightarrow \equiv {\text{LH}}$$

1 $${\text{K}}_{{{\text{a}}1}} = \frac{{\left[ { \equiv {\text{LH}}} \right]}}{{\left[ { \equiv {\text{L}}^{ - } } \right] \cdot \alpha_{{{\text{H}}^{ + } }} }}$$

R2 $$\equiv {\text{X}}^{ - } + {\text{ H}}^{ + } \leftrightarrow \equiv {\text{XH}}$$

2 $${\text{K}}_{{{\text{a2}}}} = \frac{{\left[ { \equiv {\text{XH}}} \right]}}{{\left[ { \equiv {\text{X}}^{ - } } \right]\cdot\alpha_{{{\text{H}}^{ + } }} }}$$

R3 $$\equiv {\text{M}}^{ - } + {\text{H}}^{ + } \leftrightarrow \equiv {\text{MH}}$$

3 $${\text{K}}_{{{\text{a}}3}} = \frac{{\left[ { \equiv {\text{MH}}} \right]}}{{\left[ { \equiv {\text{M}}^{ - } } \right] \cdot \alpha_{{{\text{H}}^{ + } }} }}$$

where square brackets denote the concentration of each surface functional group and α_H+_ the activity of protons in the solution; K_a_ values derived by Eqs. (1)–(3)are proton interaction constants that govern the adsorption and desorption of protons from each sample’s surfaces. A non-electrostatic surface complexation model (SCM) was generated using the software FITEQL 4.0 in order to calculate K_a_ values and ligand concentrations that best describe the excess charge data. The initial protonation stage was estimated based on Ref.^75^.

## Supplementary Information

Supplementary Information.

## Data Availability

All data are available in the main text or the supplementary materials.
