# Author Correction: Biofilms as agents of Ediacara-style fossilization

**DOI:** 10.1038/s41598-023-29279-4

**Published:** 2023-02-09

**Authors:** Silvina Slagter, Weiduo Hao, Noah J. Planavsky, Kurt O. Konhauser, Lidya G. Tarhan

**Affiliations:** 1 Department of Earth and Planetary Sciences, Yale University, New Haven, CT 06511 USA; 2 Department of Earth and Atmospheric Sciences, University of Alberta, Edmonton, AB T6G 2E3 Canada

Correction to: *Scientific Reports* https://doi.org/10.1038/s41598-022-12473-1, published online 23 May 2022

The original version of this Article contained an error in Figure 3, where an incorrect figure was displayed. The original Figure 3 and accompanying legend appear below.

**Figure 3:**
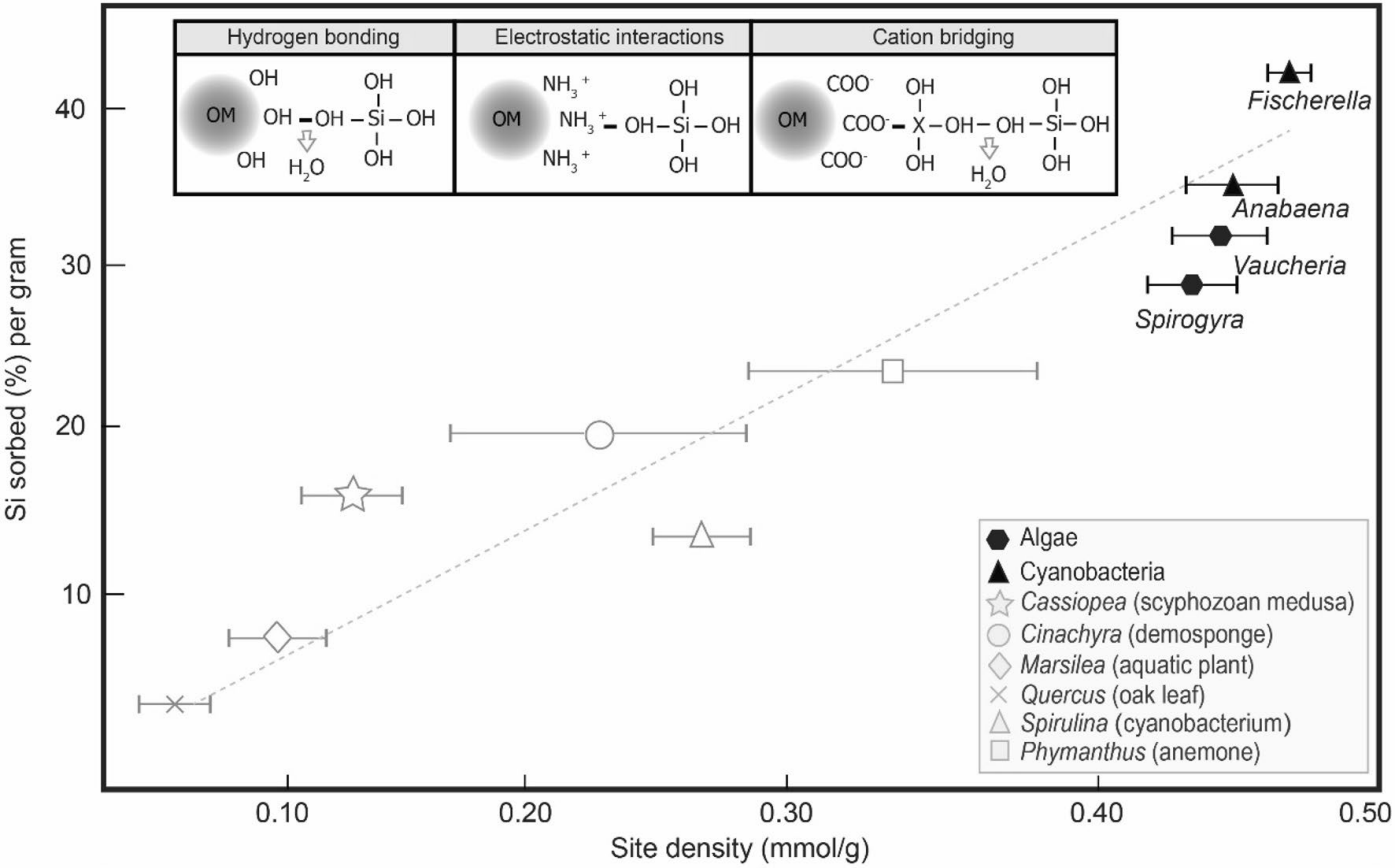
Potentiometric titration results. Mean reactive site density (mmol/g) for experimental organisms, plotted against Si absorbed, measured at the end of each experiment (150 h), and normalized to the average initial weight of each specimen. Error bars represent the standard deviation. Illustrations along the top of the panel represent the molecular bonding of silica to (from left to right) hydroxyl, amino, and carboxyl groups of organic matter (OM) (Ref.^54^). “X” represents a metal cation. Gray symbols denote data from Ref.^16^. Black symbols represent new data produced for this study. Line denotes best-fit approximation of all these data (y = 83.694x − 10.604; R^2^ = 0.7795).

Additionally, the original version of this Article contained an incomplete caption for Table 1.

“DSi dissolved silica. Biofilm refers to equal proportions of *Fischerella, Anabaena, Vaucheria, and Spirogyra*. **3-(N-morpholino)propanesulfonic acid.

now reads:

“DSi dissolved silica. Noted masses of Phymanthus are for the first run of each experiment. Biofilm refers to equal proportions of *Fischerella, Anabaena, Vaucheria, and Spirogyra*. **3-(N-morpholino)propanesulfonic acid.”

The original Article has been corrected.

