# Supplementary material for: Biofilms as agents of Ediacara-style fossilization
Source: Sci Rep. 2022 May 23;12:8631. doi: 10.1038/s41598-022-12473-1 (PMC9127100; doi:10.1038/s41598-022-12473-1)
Supplement: Supplementary file 1 — Supplementary Information. [file 41598_2022_12473_MOESM1_ESM.pdf]

Supplementary Information for

Biofilms as agents of Ediacara-style fossilization

Silvina Slagter*, Weiduo Hao, Noah J. Planavsky, Kurt O. Konhauser and Lidya G. Tarhan

*Corresponding author. Email: silvina.slagter@yale.edu

This PDF file includes:

Fig. S1. Representative photographs of the fossilization experiments.

Fig. S2. SEM-SE images of experiments associated with biofilms.

Fig. S3. Acid-base titration curves.

Fig. S4. Modeled functional groups.

Table S1. Time series of dissolved silica concentrations.

Table S2. Carbon content of experimental substrates through time.

Table S3. Silica sorbed normalized to biomass.

Table S4. Acid-base titration results and surface complexation model.

Table S5. Artificial seawater solution.

Table S6. Dissolved silica concentration in experiments with and without biofilms.

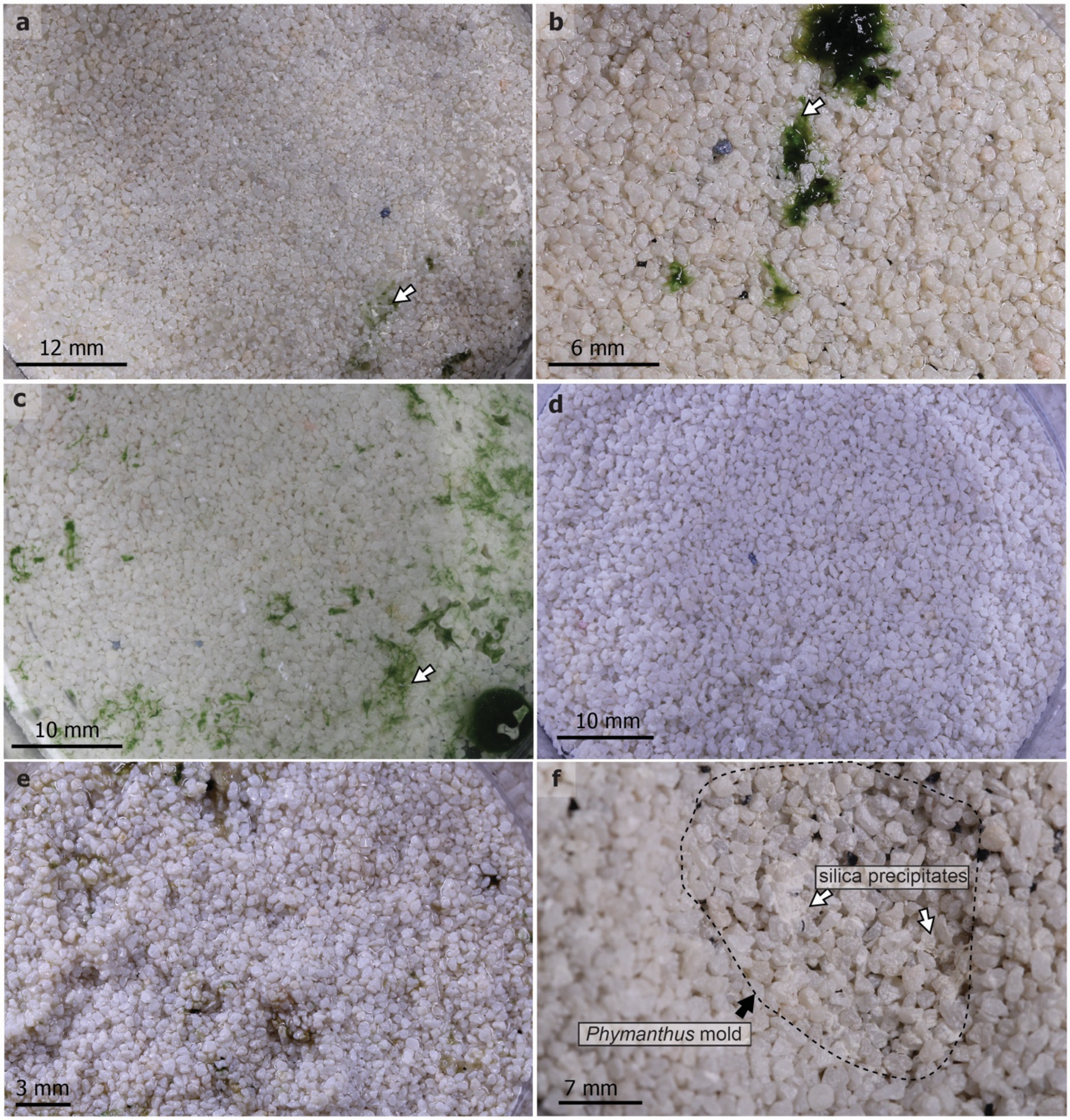

Fig. S1. Representative experimental photographs. a-d, Images of experiments after 48 hours and initial 2 mM DSi associated with *Fischerella* (experiment 4; a), *Spirogyra* (experiment 7; b), and *Anabaena* (experiment 5; c). Arrows denote examples of biofilm colonies; d-f, Images of experiments after removing *Phymanthus* (150 h), control experiment without DSi (experiment 2; d), initial 2 mM DSi (experiment 8; e) and initial 2 mM DSi and biofilms (experiment 11; f) where silica precipitates are visible in the 'proto-fossil' mold.

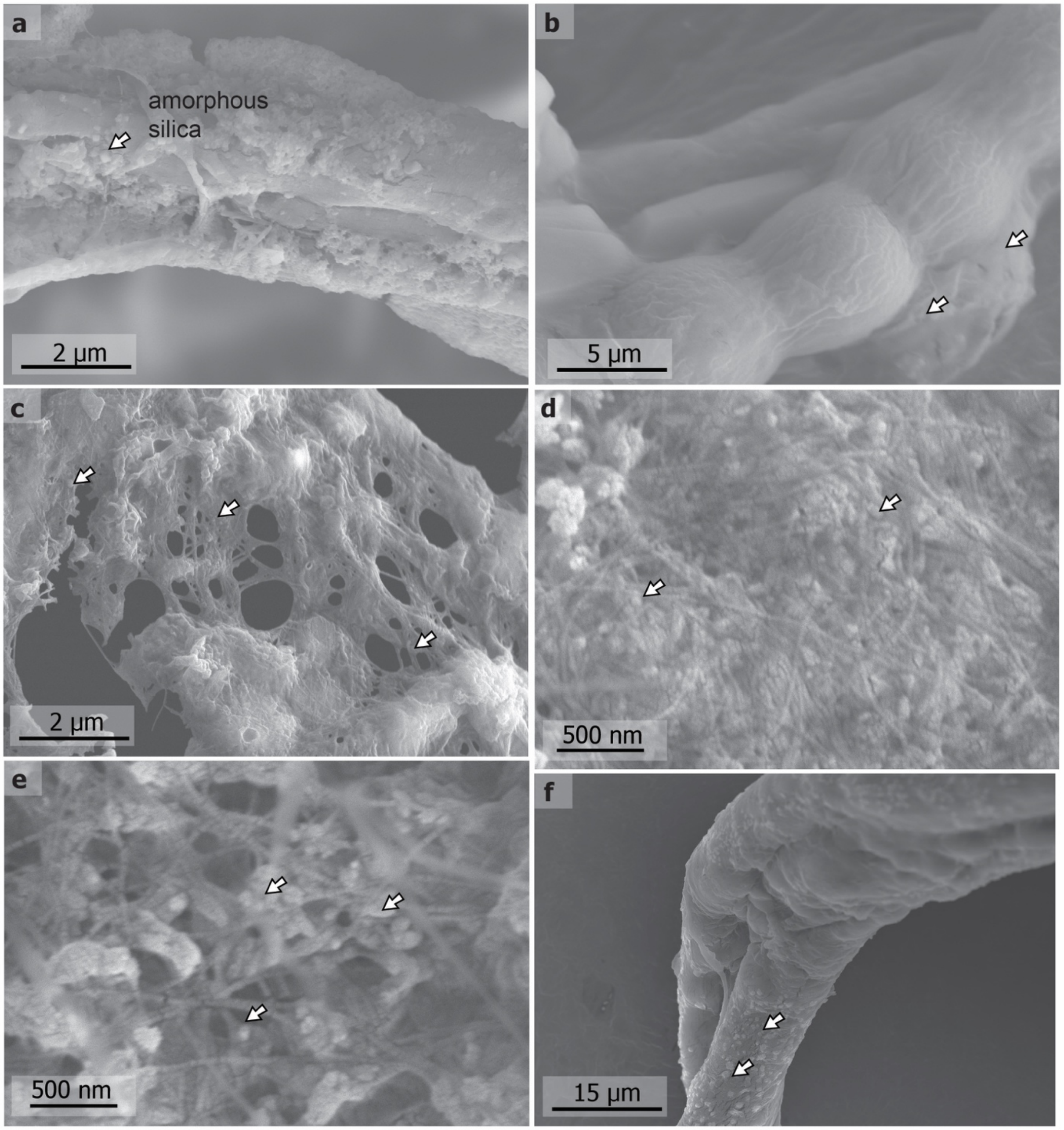

Fig. S2. SEM-SE images of experiments associated with biofilms. Images showing silica precipitation (examples indicated with arrows) associated with *Fischerella* (a; 150 h), *Anabaena* (b; 48 h), *Vaucheria* (c and d; 120 h), and *Spirogyra* (e and f; 96 h).

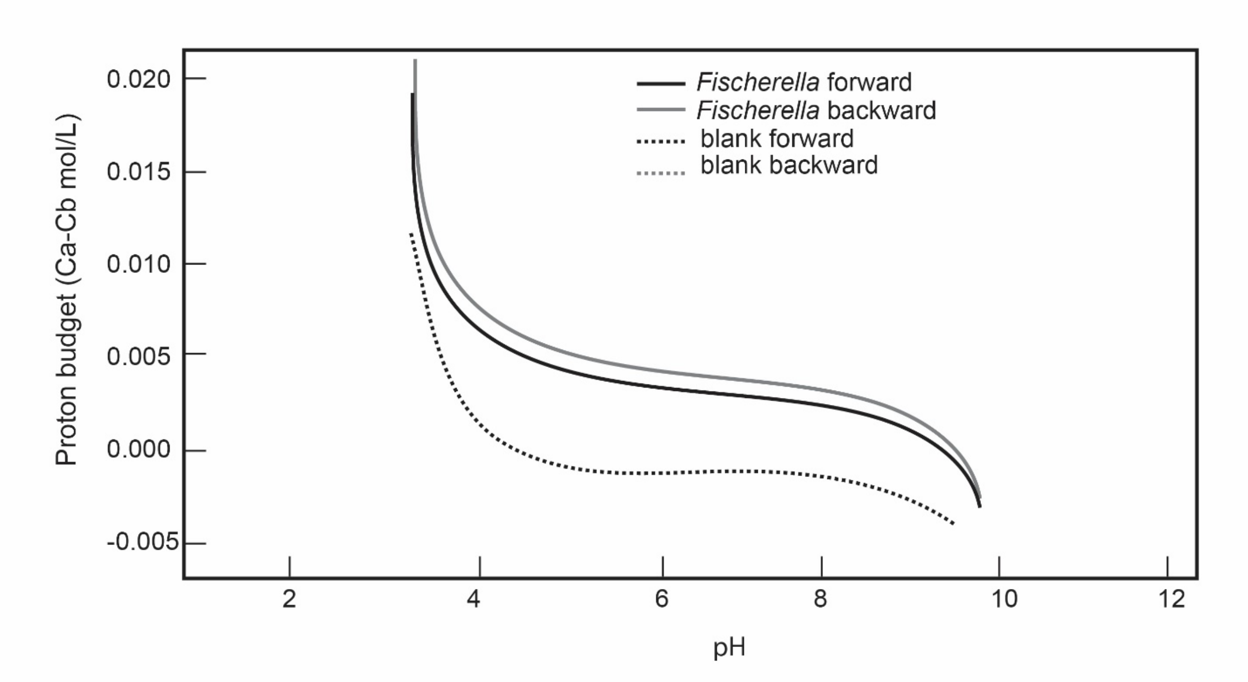

Fig. S3. Acid-base titration curves. Example of acid-base titration curves for *Fischerella.*

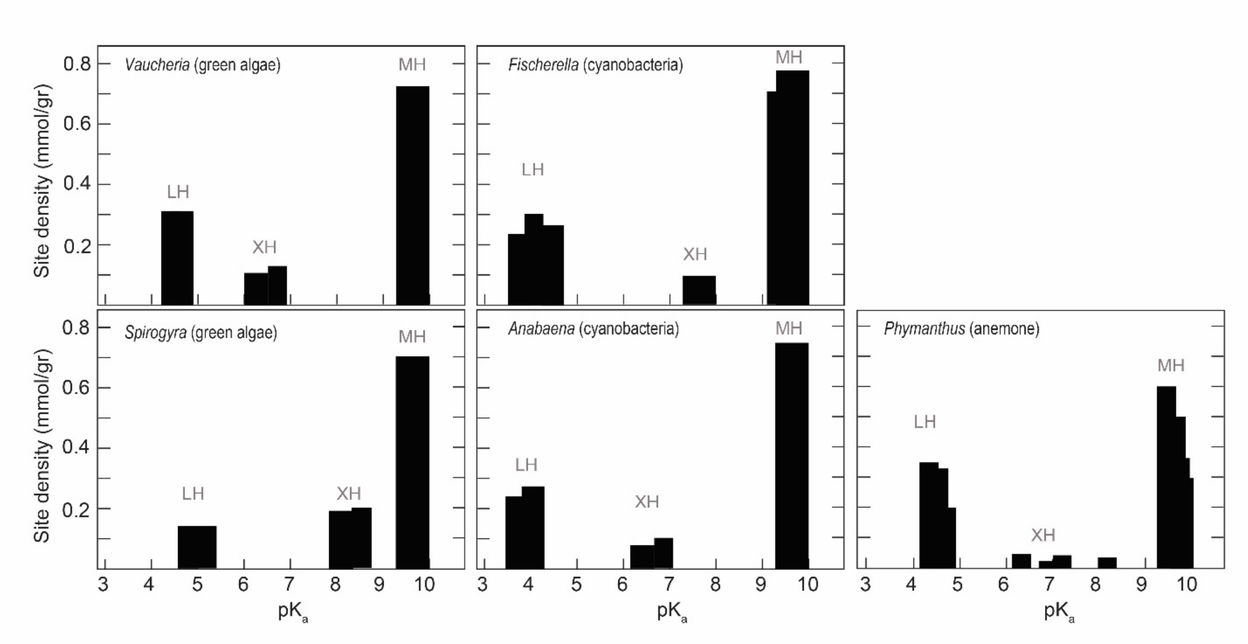

Fig. S4. Modeled functional groups, identified as ligands ≡LH, ≡XH, and ≡MH, of each experimental organism, plotted as density of deprotonated sites (excess charge) *Phymanthus* data from ref. 1.

Table S1. Time series of dissolved silica concentrations (means of three replicates)

		DSi (mM)						
	Experiment	8 h	26 h	48 h	72 h	120 h	138 h	150 h
3	DSi, Sa	2.000	1.999	1.999	1.959	1.987	2.016	1.999
4	DSi, *F*,Sa	1.550	0.630	0.589	0.525	0.515	0.450	0.439
5	DSi, *A*,Sa	1.690	0.433	0.433	0.433	0.528	0.510	0.447
6	DSi, *V*,Sa	1.849	0.442	0.531	0.348	0.584	0.784	0.436
7	DSi, *S*,Sa	1.529	1.485	1.375	1.350	1.200	0.629	0.684
8	DSi, *P*	1.014	0.880	0.665	0.699	0.631	0.753	0.677
9	DSi, *P*, Sa	1.318	0.870	0.876	0.685	0.773	0.793	0.788
10	DSi, Sa, B	1.234	0.702	0.746	0.782	0.699	0.552	0.518
11	DSi, *P*, B, Sa	1.230	0.784	0.584	0.426	0.342	0.325	0.206

DSi: dissolved silica; *P: Phymanthus,* B: biofilms, Sa: sand, *F: Fischerella*
A: Anabaena, V: Vaucheria, and *S: Spirogyra.* Experiments 1 and 2 are control experiments performed without DSi (see Main Text Table 1).

Table S2. Final dissolved silica concentration (mM) in experiments conducted both with and without biofilms

With biofilms (experiments 10 and 11)	Without biofilms (experiments 8 and 9)
0.875	1.014
0.784	0.880
0.584	0.665
0.426	0.589
0.342	0.631
0.325	0.753
0.204	0.677
0.023	1.014
0.784	0.980
0.584	0.665
0.444	0.589
0.342	0.631
0.329	1.753
0.106	0.477
0.230	0.114
0.641	1.180
0.311	1.565
0.426	0.789
0.942	0.611
0.625	0.753
0.206	0.671
0.875	1.014
0.812	0.967
0.581	0.665
0.426	0.699
0.356	0.231
0.355	0.743
0.121	0.617
0.023	1.001
0.784	1.000
0.554	0.632
0.426	0.765
0.686	0.636
0.325	1.753
1.106	0.456
0.366	0.114
0.640	1.180
0.384	1.678
0.426	0.656
0.342	0.351
0.325	0.749
0.200	0.687

Table S3. Carbon content of experimental substrates through time

Experiment	Time (h)	Weight (g)	% C
1	24	1.73	7.23
	48	1.08	2.43
	120	1.65	10.26
	144	0.45	17.54
2	72	0.31	33.79
	96	1.33	12.31
	120	0.76	9.41
	144	3.05	8.67
	150	3.8	5.4
4	24	4.8	5
	72	0.79	9.9
	96	2.9	6.5
	144	3.1	1.7
	150	3.3	0.6
5	24	2.8	5.2
	48	4.4	7.2
	120	1.2	3
	150	3.4	0.6
6	96	2.8	5
	150	5.5	2
7	48	2.19	12.1
	150	0.9	9
8	48	2.28	12.14
9	24	1.5	9.84
	150	2.7	1.8
10	48	1.2	7.23
	150	5	1.3
11	24	2.03	28.69
	72	2.29	12.75
	150	1	3.63

Table S4. Silica sorbed normalized to biomass

Experiment	Final DSi (mM)	Initial biomass (g)	Silica sorbed (mM)	Silica sorbed per gram of biomass
4	0.439	10	1.561	0.156
5	0.447	10	1.553	0.155
6	0.436	10	1.564	0.156
7	0.684	10	1.316	0.132
8	0.677	15.23	1.323	0.087
9	0.788	12.33	1.212	0.098
10	0.518	10	1.482	0.148
11	0.206	14.22	1.794	0.126

Table S5. Acid-base titration and surface complexation model results

Sample	Log(Ka)*			Site density (mol/L)**		
	Ka1	*Ka2*	*Ka3*	*LH*	*XH*	*MH*
Vaucheria	4.04 (carboxyl)	8.38 (carboxyl or phosphoryl)	10.83 (hydroxyl or amino)	3.04E-04	1.30E-04	8.02E-04
Fischerella	4.62 (carboxyl)	8.24 (carboxyl or phosphoryl)	10.8 (hydroxyl or amino)	2.52E-04	2.97E-04	9.80E-04
Anabaena	4.2 (carboxyl or phosphoryl)	7.75 (carboxyl or phosphoryl)	10.61 (hydroxyl or amino)	2.61E-04	1.22E-04	9.49E-04
Spirogyra	4.09 (carboxyl)	7.97 (carboxyl or phosphoryl)	10.36 (hydroxyl or amino)	2.22E-04	1.66E-04	7.03E-04
Phymanthus ***	5.31 (carboxyl or phosphoryl)	7.64 carboxyl or phosphoryl)	9.24 (hydroxyl or amino)	1.25E-04	4.86E-05	1.48E-04

*Identification of functional groups is based on comparison with literature[2].
**Site density and pKa (i.e., Log(Ka)) values represent the mean values of all replicates.
***Phymanthus* data from ref. 1

Table S6. Artificial seawater solution

Component	Concentration (g/kg)
NaCl	23.7034
$NaSO_4$	4.008
KCl	0.865
$NaHCO_3$	0.296
$Na_2SiO_3.9H_2O$	0.568
H_20	970.48

Supplementary references

1. Slagter, S., Tarhan, L. G., Hao, W., Planavsky, N. J. & Konhauser, K. O. Experimental evidence supports early silica cementation of the Ediacara Biota. *Geology* **49**, 51–55 (2021).

2. Fein, J. B., Boily J.-F., Yee N., Gorman-Lewis, D. & Turner, B. F. Potentiometric titrations of Bacillus subtilis cells to low pH and a comparison of modeling approaches. *Geochim. Cosmochim. Acta* **69**, 1123-1132 (2005).
